# Habitat radiomics analysis of pet/ct imaging in high-grade serous ovarian cancer: Application to Ki-67 status and progression-free survival

**DOI:** 10.3389/fphys.2022.948767

**Published:** 2022-08-25

**Authors:** Xinghao Wang, Chen Xu, Marcin Grzegorzek, Hongzan Sun

**Affiliations:** ^1^ Department of Radiology, Shengjing Hospital of China Medical University, Shenyang, China; ^2^ Department of Surgical Oncology, The First Affiliated Hospital of China Medical University, Shenyang, China; ^3^ Institute of Medical Informatics, University of Luebeck, Luebeck, Germany

**Keywords:** PET/CT, high-grade serous ovarian cancer, Ki-67, radiomics, Habitat, progression-free survival

## Abstract

**Purpose:** We aim to develop and validate PET/ CT image-based radiomics to determine the Ki-67 status of high-grade serous ovarian cancer (HGSOC), in which we use the metabolic subregion evolution to improve the prediction ability of the model. At the same time, the stratified effect of the radiomics model on the progression-free survival rate of ovarian cancer patients was illustrated.

**Materials and methods:** We retrospectively reviewed 161 patients with HGSOC from April 2013 to January 2019. 18F-FDG PET/ CT images before treatment, pathological reports, and follow-up data were analyzed. A randomized grouping method was used to divide ovarian cancer patients into a training group and validation group. PET/ CT images were fused to extract radiomics features of the whole tumor region and radiomics features based on the Habitat method. The feature is dimensionality reduced, and meaningful features are screened to form a signature for predicting the Ki-67 status of ovarian cancer. Meanwhile, survival analysis was conducted to explore the hierarchical guidance significance of radiomics in the prognosis of patients with ovarian cancer.

**Results:** Compared with texture features extracted from the whole tumor, the texture features generated by the Habitat method can better predict the Ki-67 state (*p* < 0.001). Radiomics based on Habitat can predict the Ki-67 expression accurately and has the potential to become a new marker instead of Ki-67. At the same time, the Habitat model can better stratify the prognosis (*p* < 0.05).

**Conclusion:** We found a noninvasive imaging predictor that could guide the stratification of prognosis in ovarian cancer patients, which is related to the expression of Ki-67 in tumor tissues. This method is of great significance for the diagnosis and treatment of ovarian cancer.

## Introduction

Ovarian cancer is one of the most common gynecological cancers (Torre et al., 2018). In the past few decades, although the survival rate of most tumors has improved, the 5-year survival rate of ovarian cancer has not changed since 1980 (2). Most ovarian tumors belong to high-grade serous ovarian cancer (HGSOC) (Kohn and Ivy, 2017). They usually have extensive peritoneum (III stage) or extraperitoneal (IV stage) spread in the late stage, and the risk of recurrence and death is very high (Chen and Du, 2018). In the early stages of treatment, most ovarian cancer patients respond to surgery and platinum-based chemotherapy but patients often relapse and develop resistance to chemotherapy (Jayson et al., 2014). Therefore, the exploration of prognostic biomarkers for ovarian cancer patients is constantly expanding.

Ki-67 is a kind of nuclear protein, which is expressed in the whole cell cycle of proliferating cells except for G0 cells. It is closely related to cell proliferation and invasion (Schlüter et al., 1993). In ovarian cancer, there is a clear link between Ki-67 and recurrence and prognosis of ovarian cancer (Deng et al., 2015a; Qiu et al., 2019). Positron emission tomography (PET) is a kind of functional imaging method, which can clarify the spatial distribution of the metabolic activity through tracer uptake and accurately locate the malignant lesion area combined with the anatomical information provided by CT (9). Some studies have shown that ovarian cancer PET/CT has higher preoperative staging accuracy than simple CT, and the accuracy of CT and PET/CT staging is between 53%–55% and 55%–89% (Kemppainen et al., 2019; O'Connor et al., 2013; Castellani et al., 2019). At the same time, PET/CT is more accurate in detecting recurrence than other reference standards (such as CA-125, CT, or MRI) (Limei et al., 2013). In other cancers, the radiomics model composed of noninvasive PET/ CT has had a good prediction effect, but it has not been reported in ovarian cancer (Antunovic et al., 2017; Acar et al., 2019; Kong et al., 2019). In this study, we used metabolic subregion evolution (Habitat) to improve the prediction ability of the radiomics model (Mu et al., 2020). In imaging medicine, the Habitat method is often used to divide different tumor subregions (reflecting different functional or material areas of the focus), which is a method with strong application scenarios.

## Materials and methods

### Patients

This retrospective study was approved by the review committee of our institution and was adherent to the principles and requirements of the Declaration of Helsinki. This retrospective study collected 197 patients with HGSOC in our hospital from April 2013 to January 2019. The exclusion criteria are as follows: 1) have suffered from other tumors, 2) no PET/CT scan performed, 3) any targeted treatment before scanning, and 4) performed within 3 weeks before surgery with negative 18F-FDG uptake. At the same time, the patients were operated on and treated according to NCCN guidelines. After a regular and complete follow-up (imaging data), the patients achieved progression-free survival. Progression-free survival (PFS) refers to the time from randomization to the first occurrence of disease progression or death from any cause. Finally, 161 patients were included in the study. The patients were randomly divided into a training group (*n* = 112) and test group (*n* = 49).

### 18F-FDG PET/CT acquisition

Patients were fasting from food and water for more than 6 h, and their blood sugar level was controlled below 7 mmol/L. One hour after intravenous injection of 18F-FDG (GE MINItrace II; GE Healthcare, Milwaukee, WI) at 0.08–0.16 mci/kg, PET/CT was performed from the head to the middle of the femur (GE Discovery PET/CT Elite; GE Healthcare, Milwaukee, WI). A 3dimensional PET model was used, with a matrix of 192 × 192 and an exposure time of 2 min/bed position. Low-dose spiral CT was performed at 120–140 kV and 80 ma. After CT attenuation correction, PET images were reconstructed using the algorithm of time-of-flight and point-spread-function, including 2 iterations and 24 subsets.

### Habitat generation and feature extraction

**FIGURE 1:**
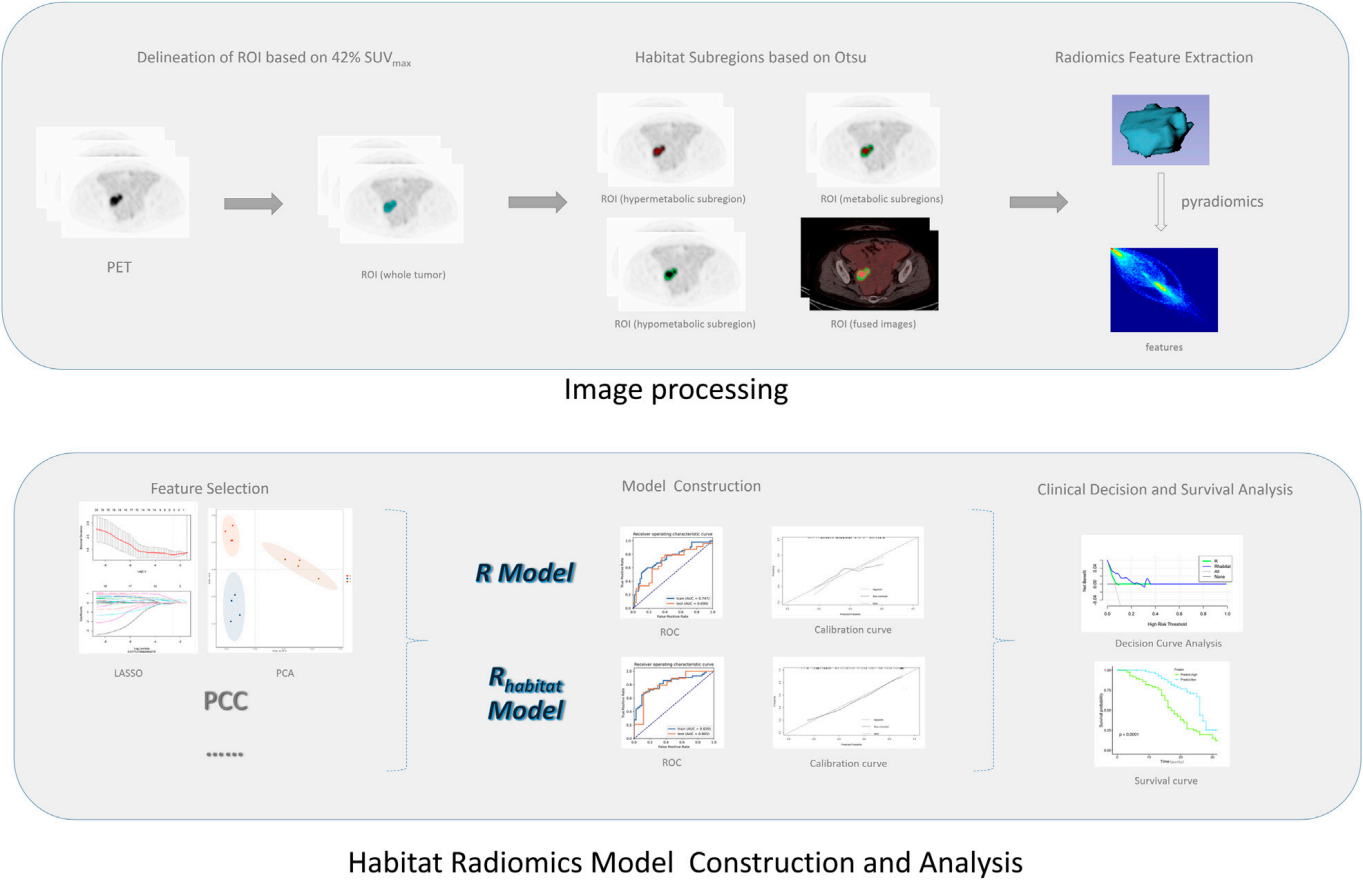
Schematic diagram of study design.

**FIGURE 2:**
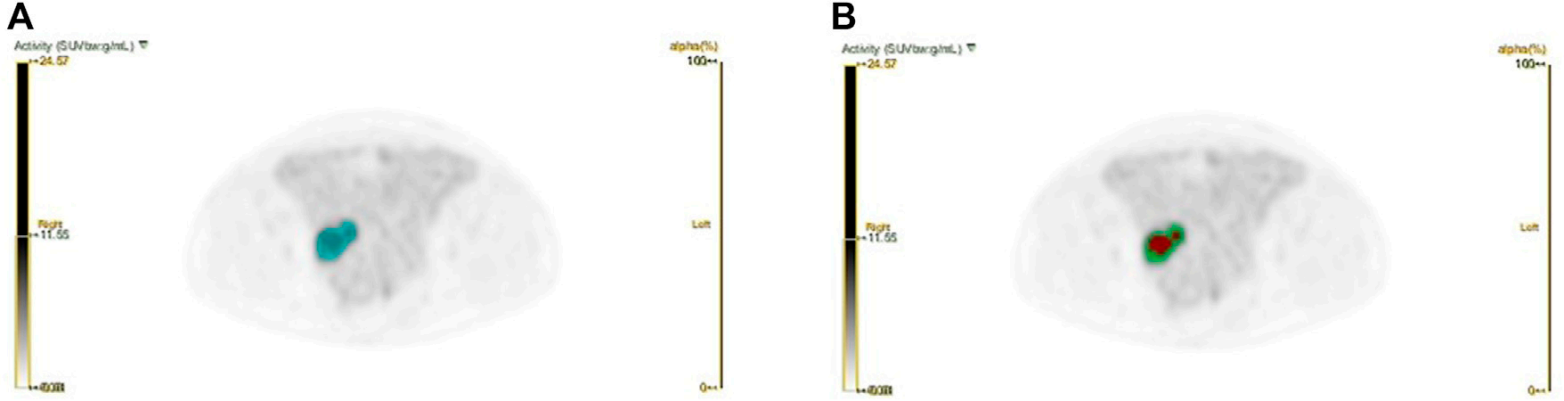
Schematic diagram of Habitat method. **(a)** The blue area represents the whole tumor area; **(b) **Based on the whole tumor area, we have implemented the subarea partition by habitat algorithm.

The workflow of radiomics is shown in Figure 1. For processing images, we have standardized processing, and then in the process of delineation and ROI processing we used LIFEx software (https://www.lifexsoft.org/) and ITK-SNAP (http://www.itksnap.org/pmwiki/pmwiki.php). Based on the metabolic threshold of PET images, we quickly identified the tumor contour. On the python (version 3.8.5) platform, we implemented the Otsu threshold way by self-built code, and we obtained two metabolic subgroups that maximized the variance between groups. Based on the threshold, the corresponding tumors in PET images were divided into the high metabolism region (the red region in Figure 2) and the low metabolism region (the green region in Figure 2), representing different Habitat subgroups. We define the difference in SUV metabolism between these two subregions, and we define them as PET_hight_ and PET_low_. So far, we have obtained three kinds of ROIs based on PET images, including the whole tumor. Based on each ROI and its effect on the image, we extract 1316 texture features, replace the abnormal value of omics features with the average value, and then separate the feature data according to the average value *μ* = 0 δ 2 = 1.

### Feature selection and model establishment

For the selection of models, we adopt a variety of methods, such as PCC, PCA, and Lasso, to operate separately or in parallel many times, in order to remove redundant and strongly collinear or correlated variables, reduce model parameters, make them match the sample size of this study, and avoid overfitting or underfitting phenomenon in the final prediction model.

For modeling, we use common classifiers (SVM, LAD, logistic regression, decision tree, random forest, and the naive Bayesian algorithm). In different models, the model with high AUC and strong generalization ability is selected to be included in the final model selection. According to the different ROI extracted, the feature extracted from the whole tumor region is called R, and the model based on the Habitat region (PET_hight_ and PET_low_) extraction is R_habitat_.

### Statistical analysis

SPSS statistical software (version 24.0; IBM), R (version 3.63), and MedCalc Statistical Software version 15.2.2 (MedCalc Software bvba, Ostend, Belgium; http://www.medcalc.org; 2015) were used for all analyses. The clinical characteristics of the training group and the validation group were statistically tested to test their data distribution. The *t* test was used for data with normal distribution and homogeneity of variance, and the *U* test was used for data without normal distribution. The Delong test was performed on different models in the training group and the test group. Clinical decision curves and survival curves of different group models were compared to explore the significance of PFS.

## Results

### Clinical features

The clinical characteristics of patients in the training group and the validation group are shown in Table 1. There was no significant difference in clinical characteristics between the two groups.

**TABLE 1:** Clinical characteristics of HGSOC patients in training and test groups.

Characteristic	Training group (n = 112)	Test group (n = 49)	*p* value
Age, mean ± SD, year	53.22±9.31	53.43±10.52	*p* > 0.1
NACT			*p* > 0.1
Yes	43	13	
No	69	36	
LNM			
Yes	59	29	*p* > 0.1
No	53	20	
FIGO stage			*p* > 0.1
Stage III	72	33	
Stage IV	40	16	
Ascites			*p* > 0.1
<200 ml	61	24	
200ml–1000 ml	37	19	
>1000 ml	14	6	

### Description and comparison of prediction models

**FIGURE 3:**
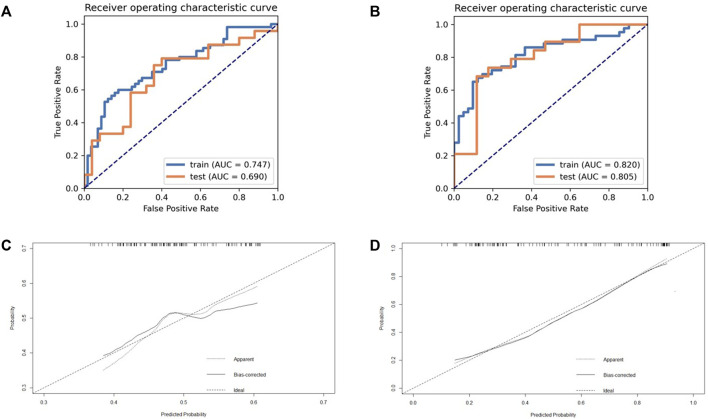
The ROC and calibration curve of R and R_habitat_. **(a)** The performance of training set and validation set on model R (based on whole tumor); **(b)** The performance of training set and validation set on model R_habitat_ (based on different metabolic sub-regions). **(c)** Demonstrate the calibration effect of the R model; **(d)** Demonstrate the calibration effect of the R_habitat_ model.

The model takes the patient’s Ki-67 status (>50%) (Qiu et al., 2019) as the label for modeling and analysis. The details of R and R_habitat_ models generated are shown in Table 2. According to the diagnostic efficiency, we found that the R_habitat_ model (the training group: the AUC value is 0.835, 95% CIs: [0.7240-0.9460], accuracy: 0.7919, sensitivity: 0.8377; the training group: the AUC value is 0.835, 95% CIs: [0.7240-0.9460], accuracy: 0.7919, sensitivity: 0.8377; the training group: the AUC value is 0; the test group: AUC = 0.8076, 95% CIs: [0.7225-0.9611], accuracy: 0.7557, sensitivity: 0.8192) had the highest diagnostic efficiency (Figure 3), higher than R (the training group: AUC = 0.7670, 95% CIs: [0.6842-0.8498], accuracy: 0.7519, sensitivity: 0.8077); the test group: the AUC value was 0.7488, 95% CIs: [0.6583-0.8393], accuracy: 0.7223, sensitivity: 0.7892). In the Delong test, we found that the efficiency of the R_habitat_ model was higher than that of the R model (*p* < 0.05).

**TABLE 2:** Description of two models.

Model	Standardization method	Feature selection method	Characteristic quantity	Model classifier
**R**	Z-score	Recursive feature elimination	20	Auto-encoder
**R**_**habitat**_	Z-score	Kruskal–Wallis	8	Logistic regression

### Decision curve analysis and survival analysis

**FIGURE 4:**
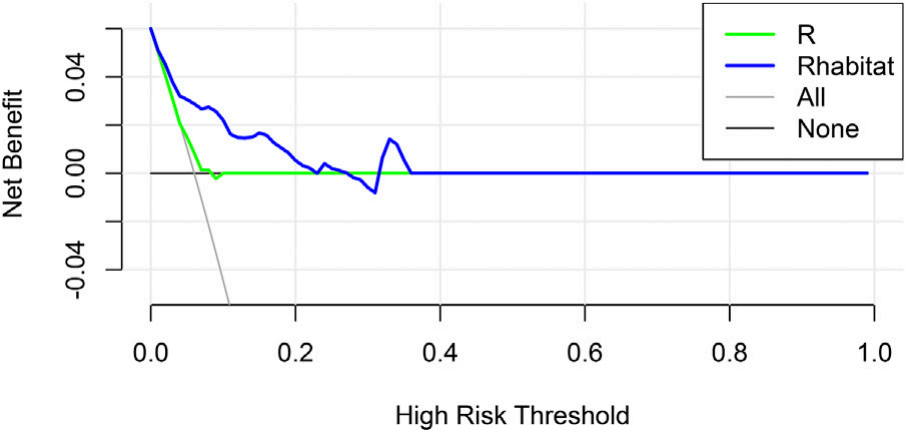
Decision curve analysis for R and R_habitat_.

The decision curve analysis (DCA) displays estimates of a series of probability threshold (normalized) net benefits used to classify observations as “high risk.” These curves help to assess a treatment policy that recommends that the impact of a risk-based policy on the population be compared with the “treat all” and “no treat” intervention policies, thereby recommending treatment for patients estimated to be “at high risk.” DCA of the two models is shown in Figure 4.

**FIGURE 5:**
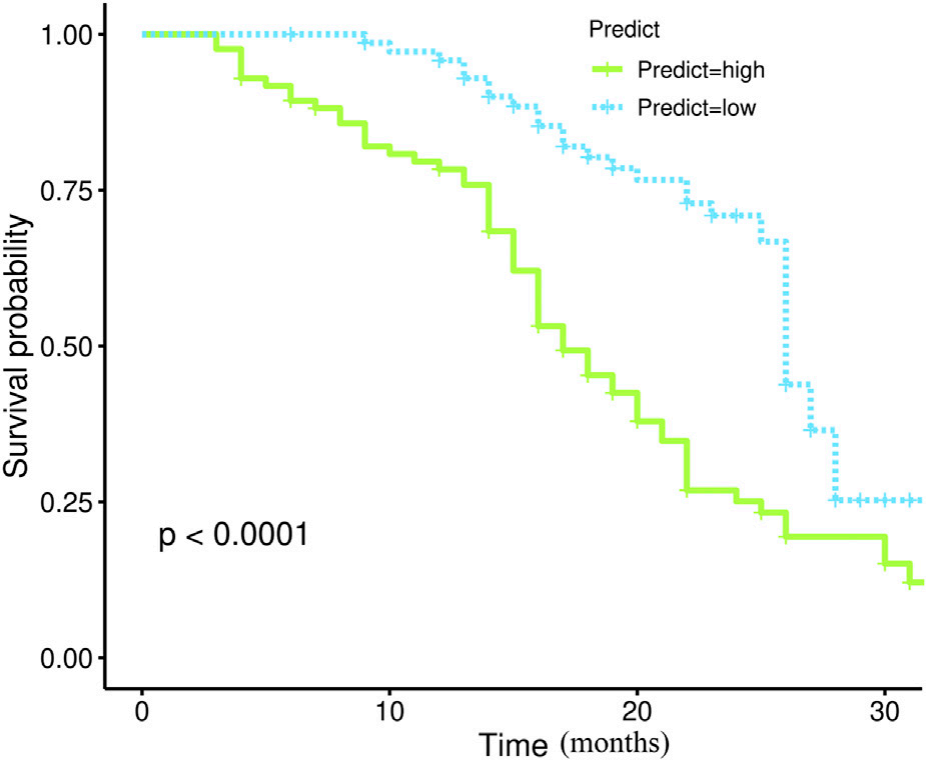
The survival curves of R_habitat_.

Through the aforementioned screening, we obtained the combination model R_habitat_ with strong classification and prediction ability. In order to further explore its prediction ability for the prognosis of patients with HGSOC, we drew the survival curve and found that the model has a strong prediction stratification ability, and the K-M test (Figure 5) has a significant difference (*p* < 0.001).

## Discussion

In this study, we developed and validated radiomics from positron emission tomography (PET), computed tomography (CT), and the Habitat subregion to predict the Ki-67 status in patients with ovarian cancer and to explore its role in prognostic stratification. Patients can be divided into low-risk and high-risk groups through the establishment of an imaging omics model. There is a significant difference between the model using the Habitat subregion and the traditional model.

In recent years, some scholars have proposed the molecular classification of ovarian cancer related to prognosis, which is marked as differentiation, immunoreactivity, mesenchymal, and proliferative (Tothill et al., 2008; Verhaak et al., 2013). At the same time, studies have shown that the molecular classification based on CT imaging features can effectively distinguish ovarian cancer and can be used as a predictor of prognosis (Vargas et al., 2015). A multicenter study used computed tomography imaging features to assess its association with disease progression time and ovarian cancer transcriptomic characteristics and to develop an image-based risk scoring system (Vargas et al., 2017). Studies have also been conducted to explore the association between proteomics and imaging omics. It is found that four proteins in ovarian cancer patients are related to CT-based imaging. Among them, the correlation between the CRIP2 protein and mesenteric diseases is strongest, and the abundance of other three proteins (STXVP2, ASS1, and CBD) is related to the heterogeneity of tumor location (Beer et al., 2020). There are a lot of research on prognosis but there are some problems of insufficient explanation, such as trying to find the interpretable aspect of gene transcription (Lu et al., 2019). Some studies suggest that SUV_max_ and SUV_mean_ are moderately correlated with the Ki-67 index, which confirms the value of the PET image for Ki-67 but it is difficult to accurately predict the expression of Ki-67 (Deng et al., 2015b; Mayoral et al., 2018. We use the Habitat method to generate two metabolic subregions with different metabolic characteristics (Mu et al., 2020). In this experiment, the Otsu scheme is used. The principle is to generate two parts so that the overall similarity of each part is the highest and the difference between different parts is the largest. Automatic segmentation is realized by a variance correlation algorithm. At present, the Habitat method has been applied in many medical images. Some studies have shown that it can significantly improve the performance of PFS and OS models in predicting locally advanced cervical cancer patients in PET/ CT (16) and has a significant predictive value for glioma prognosis (Verma et al., 2020; Park et al., 2021), nasopharyngeal carcinoma (Xu et al., 2020), lung cancer (Cherezov et al., 2019), and prostate cancer (Parra et al., 2019). For this research model, the overall characteristics of the tumor were eventually incorporated into the PET mode high metabolic area and low metabolic area, where the high metabolic area represented the strongest part of the tumor activity, which had a significance for the prognosis of patients (Pinho et al., 2020), while the PET low metabolism area was often the edge of the tumor and correlated with immune infiltration (Grove et al., 2021). At the same time, we should note that Habitat subregions generated by PET modality are more heterogeneous and eccentric, and are often associated with poor prognosis (Mu et al., 2020). We used the random grouping method to ensure the independence of data between the training group and test group, and verified by the test group, which showed that the PET/MR mode radiology model based on the Habitat method has some generalization ability. We have also noted that some scholars used enhanced CT scanning images to process images with the Gaussian mixture model (another common analysis method in the field of habitat analysis) to identify cystic and solid tumor subregions and help ovarian cancer patients with accurate puncture (Beer et al., 2021). The Gaussian mixture model could aim to distinguish the heterogeneity of mixture since its birth, which was undoubtedly in line with the application background of the aforementioned research. Just as the Gaussian mixture model could better distinguish the cystic and solid parts of ovarian cancer, our research used the Otsu model with maximized variance between groups to identify areas with more active metabolism to represent the tumor proliferation activity (Ki-67) and prognosis. Our outlook for this technology provides a noninvasive method to evaluate the activity of local lesions in chemotherapy for patients with high-grade serous ovarian cancer, which is also related to the overall survival time of patients.

At the same time, this study has the following shortcomings (Torre et al., 2018): a single center, that is, the lack of effective external verification, will undoubtedly greatly affect the generalization and application ability of the model (Lisio et al., 2019); the PET modal images processed by Habitat have been verified by the literature, but CT images were not included in this study due to cautious attitude and early development (Kohn and Ivy, 2017). For the study of PFS, there is a lack of further involvement of clinical factors, which needs further multicenter and large-scale data research.

## Conclusion

Noninvasive imaging prediction indicators based on PET images can guide the prognosis stratification of ovarian cancer, which is related to the expression of Ki-67 in tumor tissues, and the accuracy of Habitat is improved. In the diagnosis and treatment of ovarian cancer, it is important to use a variety of technical means to guide the prognosis and molecular typing, especially for noninvasive means.

## Data Availability

The raw data supporting the conclusion of this article will be made available by the authors, without undue reservation.
